# Whole genome survey analysis and microsatellite motif identification of *Sebastiscus marmoratus*

**DOI:** 10.1042/BSR20192252

**Published:** 2020-02-24

**Authors:** Sheng-yong Xu, Na Song, Shi-jun Xiao, Tian-xiang Gao

**Affiliations:** 1 Fishery College, Zhejiang Ocean University, Zhoushan 316022, P.R. China; 2 Institute of Evolution and Marine Biodiversity, Ocean University of China, Qingdao 266003, P.R. China; 3 School of Computer Science and Technology, Wuhan University of Technology, Wuhan 430070, P.R. China

**Keywords:** genome size, genome survey, marbled rockfish, microsatellite marker

## Abstract

The marbled rockfish *Sebastiscus marmoratus* is an ecologically and economically important marine fish species distributed along the northwestern Pacific coast from Japan to the Philippines. Here, next-generation sequencing was used to generate a whole genome survey dataset to provide fundamental information of its genome and develop genome-wide microsatellite markers for *S. marmoratus*. The genome size of *S. marmoratus* was estimated as approximate 800 Mb by using K-mer analyses, and its heterozygosity ratio and repeat sequence ratio were 0.17% and 39.65%, respectively. The preliminary assembled genome was nearly 609 Mb with GC content of 41.3%, and the data were used to develop microsatellite markers. A total of 191,592 microsatellite motifs were identified. The most frequent repeat motif was dinucleotide with a frequency of 76.10%, followed by 19.63% trinucleotide, 3.91% tetranucleotide, and 0.36% pentanucleotide motifs. The AC, GAG, and ATAG repeats were the most abundant motifs of dinucleotide, trinucleotide, and tetranucleotide motifs, respectively. In summary, a wide range of candidate microsatellite markers were identified and characterized in the present study using genome survey analysis. High-quality whole genome sequence based on the “Illumina+PacBio+Hi-C” strategy is warranted for further comparative genomics and evolutionary biology studies in this species.

## Introduction

The assessment of genetic diversity and structure is one of the major goals of population management and conservation biology [1]. This assessment should ideally be achieved by utilizing polymorphic and informative markers. Microsatellites or simple sequence repeats (SSRs) are short tandem repeated motif (1–6 bases) that are found in both non-coding and coding regions of the genome and are characterized by a high degree of length polymorphism [2]. Microsatellite markers have become one of the most popular molecular markers and have been widely used in genetic studies due to their ubiquitous occurrence, high reproducibility, multiallelic nature, and codominant mode [2,3]. The advantages of microsatellite markers have made them one of the most useful tools for detecting genetic diversity, genetic linkage mapping, genetic structure, and germplasm and evolution analysis. However, conventional approaches to isolate and develop microsatellite primers were time- and cost-consuming because it is necessary to create enriched microsatellite libraries [2]. Until recently, next-generation sequencing (NGS) has provided a new perspective for the development of studies of microsatellite markers, owing to its high throughput and speed of data generation. So far, NGS has been applied to genomics-based strategies to discover sequences for new microsatellite markers in animals and plants, in a time- and cost-effective manner [4–8]. Genome survey sequencing (GSS) based on the NGS platform has been proven particularly useful in identifying genome-wide microsatellite markers in non-model species. Microsatellite markers development studies from GSS were performed in numbers of species [9–13]. Genome survey studies also provide information about genome structure of organisms, including estimates of genome size, levels of heterozygosity, and repeat contents.

The marbled rockfish (*Sebastiscus marmoratus*, Cuvier, 1829) is an ecologically and economically important ovoviviparous marine species inhabiting littoral rocky bottoms along the northwest Pacific coast from Japan to the Philippines [14]. *S. marmoratus* has strong site fidelity and appears within narrow home ranges [14]. Several studies have been conducted on *S. marmoratus* germplasm resources due to the decline in wild populations [15–17]. However, inconsistent results were demonstrated given the insufficient resolution of molecular markers. Till now, limited microsatellite marker resources are publically available for *S. marmoratus* using different methods [18–22]. The use of microsatellite markers in molecular studies is limited and more microsatellite markers are needed for further studies. In the present study, we aimed to characterize and develop genome-wide microsatellite markers in *S. marmoratus* by genome survey sequencing. The newly identified microsatellites would be useful for extending our current knowledge of *S. marmoratus* genome organization and for genome mapping, marker-aided selection, and population genetics.

## Materials and methods

### Sample collection and genome survey sequencing

One male adult *S. marmoratus* was collected from Rushan (36°43′N, 121°39′E), China in October 2015. Muscle tissue was stored in 95% ethanol at −80°C. Total genomic DNA was extracted using a standard phenol–chloroform method for muscle tissue. DNA was treated with RNase A to produce pure, RNA-free DNA. Two paired-end DNA libraries were constructed with insert size of 350 bp, and then sequenced using the Illumina HiSeq2500 platform following the manufacturer’s protocol. The library construction and sequencing were performed at Novogene in Beijing.

### Data analysis

After removing low quality reads, all clean data were used to perform K-mer analysis. Based on the results of the K-mer analysis, information on peak depth and the number of predicted best K-mer were obtained and used to estimate the size of the genome. Its relationship was expressed by using the following algorithm: Genome size = K-mer_num/peak_depth, where K-mer_num is the total number of predicted best K-mer, and peak_depth is the expected value of the K-mer depth. Also, the heterozygosity ratio and repeat sequence ratio were estimated following the description in [23], based on the K-mer analysis. K-mer analyses were performed using software GCE v1.0.0 [24] and KmerGenie v1.7039 [25], respectively. The clean reads were assembled into contigs in software SOAPdenovo v2.01 [26] with a K-mer of 21 by applying the *de Bruijn* graph structure. The paired-end information was then used to join the unique contigs into scaffolds.

The Perl script MIcroSAtellite (MISA, http://pgrc.ipk-gatersleben.de/misa/) was used to identify microsatellite motifs in the *de novo* draft genome. The search parameters were set for the detection of di-, tri-, tetra-, penta-, and hexanucleotide microsatellite motifs with a minimum of 6, 5, 5, 5, and 5 repeats, respectively. The microsatellite loci were subjected to primer design using Primer3 v2.3.7 software [27,28] with the standard parameters.

## Results and discussion

### Genome size prediction and sequence assembly

**Figure 1:**
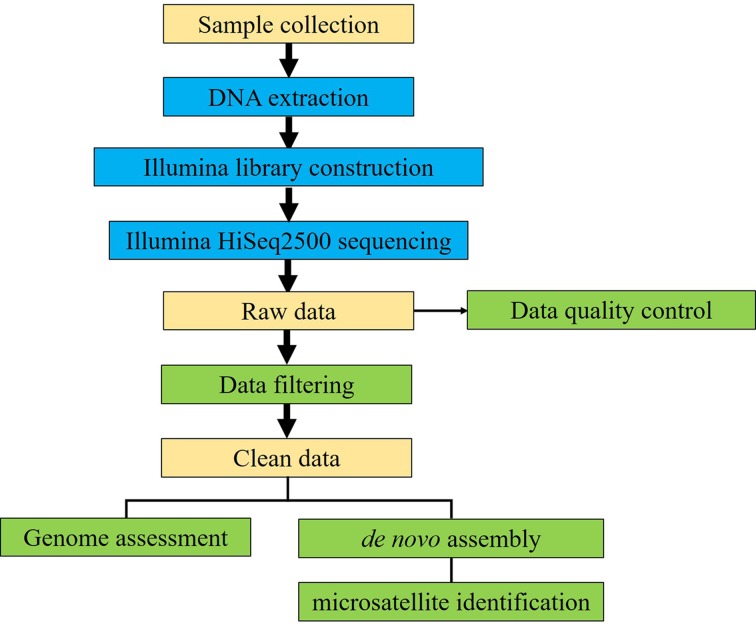
Overview of the experimental design and analysis pipeline

**Figure 2:**
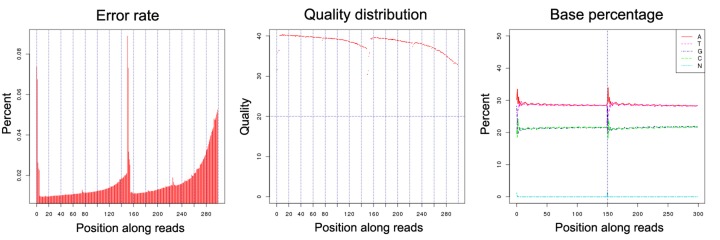
Distribution figure of error rate, sequencing quality and GC content of raw data

**Figure 3:**
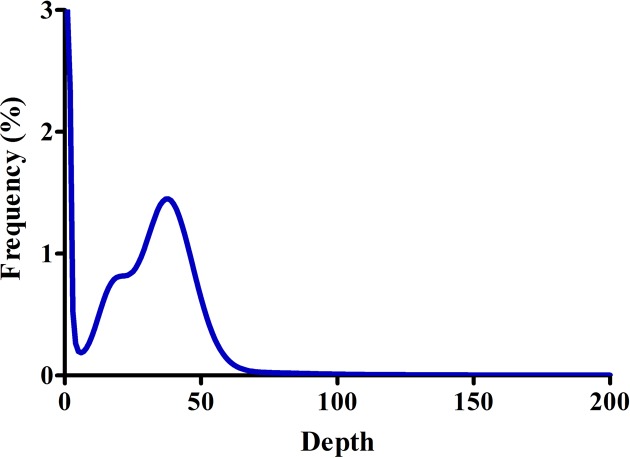
K-mer (21-mer) analysis for estimating the genome size of *S. marmoratus* The *X*-axis is depth and the *Y*-axis is the proportion that represents the frequency at that depth. Data produced from 350 bp insert library. The peak K-mer frequency was 38.

The experimental design, sequencing and analysis pipeline is shown in Figure 1. A total of 35.1 Gb raw data were generated by sequencing genome survey library with 350 bp inserts. The effective rate, error rate, Q20, Q30, and GC content of raw data was shown in Table 1 and Figure 2. A total of 34.8 Gb clean data were obtained after filtering and used for K-mer analysis. When employing KmerGenie, the predicted best K for K-mer analysis was 107 and the predicted genome size was about 812.86 Mb. Comparatively, when using GCE, the 21-mer frequency distribution derived from the sequencing reads is plotting in Figure 3; the peak of the 21-mer distribution was 38, and the total K-mer count was 29,998,886,801. As a result, the genome size was estimated as 796.25 Mb and the heterozygosity ratio and repeat sequence ratio were 0.17% and 39.65%, respectively. The development of NGS technology has provided researchers with an affordable way of addressing a wide range of questions, especially in non-model species such as *S. marmoratus* [11]. In addition, the K-mer method has been successfully applied for the estimation of genome size using NGS reads without prior knowledge of the genome size [29]. Here, for the first time, we reported a genome survey of *S. marmoratus* using whole genome shotgun sequencing. The K-mer analyses suggested that the genome size is about 800 Mb, which is 87% of the size (920 Mb) previously estimated for *S. marmoratus* using flow cytometry [30].

**Table 1:** Quality control information of Illumina sequencing data

Lib ID	Raw data (bp)	Clean data (bp)	Effective rate (%)	Error rate (%)	Q20	Q30	GC content (%)
DES_L5	35,057,094,600	34,843,246,022	99.39	0.02; 0.03	98.02; 95.13	94.91; 89.18	42.86; 43.04

Note: The two statistics of error rate, Q20, Q30, and GC content were for pair-end read 1 and read 2, respectively.

Assembly was performed using 34.8-Gb Illumina PE clean reads. The length of contig N50 was 674 bp, and the Scaffold N50 was 4362 bp. The total length of scaffolds was 609.46 Mb. The GC content of scaffolds was 41.3%. The number of scaffolds >100 bp was 412,901 (98.99%) and >1 kb was 188,316 (45.15%) (Table 2). Information about the genome size of *S. marmoratus* from the present study may be useful for further genomic studies in this species.

**Table 2:** The result of assembly in *S. marmoratus* using 34.8-Gb Illumina clean data

	Contigs		Scaffolds	
	Size (bp)	Number	Size (bp)	Number
N90	145	995,699	1117	179,431
N80	241	680,067	1877	128,640
N70	373	485,655	2589	94,551
N60	518	353,199	3413	69,208
N50	674	254,586	4362	49,699
Total size	583,830,195	–	609,456,819	–
GC content	41.39%	–	41.30%	–
Total number (>100 bp)	1,467,661		412,901	
Total number (>1 kb)	127,823		188,316	

### Identification and characteristic of microsatellite motifs in genome survey

**Figure 4:**
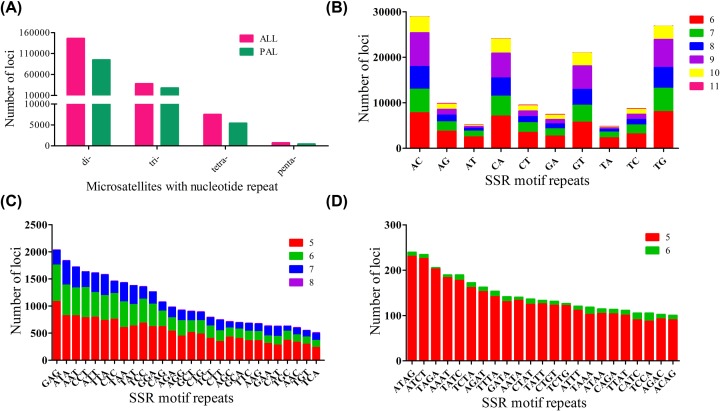
The distribution and frequency of microsatellite motifs (**A**) Frequency of different microsatellite repeat types. ALL, all of the identified microsatellites, PAL, potentially amplifiable loci. (**B**) Frequency of different dinucleotide microsatellite motifs. (**C**) Frequency of different trinucleotide microsatellite motifs. (**D**) Frequency of different tetranucleotide microsatellite motifs.

From the 609,456,819 bp genome survey sequence, a total of 191,592 microsatellite motifs were identified, which included 140,801 microsatellite-containing sequences. However, only 67,846 sequences contained more than one microsatellite motifs, and 16,325 microsatellites were present in compound formation. Therefore, the microsatellite distribution frequency in this genome was estimated to be about 314.6 microsatellite per Mb. The motif types of microsatellites included 76.10% dinucleotide, 19.63% trinucleotide, 3.91% tetranucleotide, 0.36% pentanucleotide, and few hexanucleotide repeats (Figure 4A, Supplementary Table S1). The number of dinucleotide repeats was the highest, which was similar to previous studies on the distributions and characteristics of the microsatellites in *S. marmoratus* [22]. The frequency of repeats in most eukaryotes decreases exponentially with repeat length because mutation rates are higher in longer repeats [31]. Chen et al. [32] also reported that the number of repeats is inversely correlated with repeat length, and our present results confirmed this pattern. The relative abundances of specific repeat motifs were highly variable among the repeats. The frequency distribution range of microsatellite repeats ranged from 6 to 11 repeats for dinucleotide, from 5 to 8 repeats for trinucleotide, from 5 to 6 repeats for tetranucleotide. Of the dinucleotide repeats, the AC, TG, CA, and GT repeats were the first four repeats in abundance, accounting for 19.8% (28,853), 18.4% (26,797), 16.5% (24,093), and 14.3% (20,896), respectively (Figure 4B). Of the trinucleotide repeats, the GAG repeat was the most abundant, accounting for 5.4% (2030), whereas the ACG repeat was the least, accounting for 0.07% (25). In terms of the frequency of repeats, the 5-fold repeat was the most frequent of all trinucleotide repeats (Figure 4C). Of the tetranucleotide repeats, the ATAG repeat was the most abundant, accounting for 3.2% (239) (Figure 4D). However, only 5- and 6-fold repeats were identified and the 5-fold repeat was predominant of all tetranucleotide repeats. Compared with the results of Song et al. [22], in which the distributions and characteristics of the microsatellites in *S. marmoratus* were analyzed on the basis of 454 FLX pyrosequencing technique, our results showed high-efficiency in microsatellite loci identification. The number of microsatellites in the present study, as well as the kinds of microsatellite motifs, was much higher than previous study using 454 FLX pyrosequencing technique [22]. This difference might be due to the higher throughput of Illumina sequencing than 454 pyrosequencing.

In the present study, primers were designed for the di- to pentanucleotide repeats to develop genome-wide microsatellite markers in *S. marmoratus*. With the exceptions of compound repeats, primers were successfully designed for 65.43%, 73.40%, 72.15%, and 63.45% of the di-, tri-, tetra-, and pentanucleotide loci, respectively, proving themselves to be promising candidates for PCR amplification (Figure 4A).

Genomic microsatellite markers, which are reliable, highly polymorphic, multi-allelic, and easy to amplify, are widely used in population genetics, linkage analysis, evolutionary studies and so on [33]. Queirós et al. [34] suggested that reliable and accurate estimates of genetic diversity can be obtained using random microsatellites distributed throughout the genome because selecting the most polymorphic markers will generally overestimate parameters of genetic diversity, leading to misinterpretations of the actual genetic diversity, which is particularly important for managed and threatened populations. In the present study, we provided various candidate genomic microsatellites for *S. marmoratus* that will enhance the range of markers for this species after amplification and testing in various populations. This is the first study to analyze the genome size and the characteristics of *S. marmoratus* microsatellites using genome survey sequencing. The results will be helpful for future population genetics and germplasm resource conservation. In addition, we suggested further studies should generate high-quality whole genome sequence of *S. marmoratus* based on the combination of “Illumina+PacBio+Hi-C” techniques, to provide robust information for genomic and evolutionary biology studies.

## Supplementary Material

Supplementary Table S1

## Abbreviations

GSS: Genome survey sequencing
NGS: next-generation sequencing
SSR: simple sequence repeat
