# Supplementary material for: Whole genome survey analysis and microsatellite motif identification of Sebastiscus marmoratus
Source: Biosci Rep. 2020 Feb 24;40(2):BSR20192252. doi: 10.1042/BSR20192252 (PMC7040462; doi:10.1042/BSR20192252)
Supplement: Supplementary Table S1 [file BSR-2019-2252_supp.pdf]

Table S1 Frequency of identified SSR motifs

Repeats	5	6	7	8	9	10	11	12	Total
AC	-	7749	5192	4967	7437	3450	58		28853
AG	-	3691	2063	1473	1287	1100	193		9807
AT	-	2457	1252	627	374	222	134	9	5075
CA	-	7026	4355	4029	5456	3103	70		24039
CG	-	37	14	1					52
CT	-	3453	2100	1354	1242	1128	165		9442
GA	-	2646	1585	1086	961	937	166		7381
GC	-	32	7	3	1				43
GT	-	5697	3700	3486	5172	2798	43		20896
TA	-	2249	1256	601	328	200	127	8	4769
TC	-	3111	1958	1208	1146	1053	172		8648
TG	-	8000	5144	4562	6175	2885	31		26797
AAC	327	135	124	13					599
AAG	355	169	137	11					672
AAT	814	512	384	6					1716
ACA	255	102	115	11					483
ACC	87	53	27	2					169
ACG	17	4	3	1					25
ACT	111	63	56	3					233
AGA	533	245	188	11					977
AGC	421	168	104	15					708
AGG	441	283	185	12					921
AGT	145	72	50	10					277
ATA	817	565	442	10					1834
ATC	171	95	118	15					399
ATG	177	104	133	11					425
ATT	788	454	357	9					1608
CAA	193	96	61	13					363
CAC	192	86	44	8					330
CAG	614	286	163	8					1071
CAT	274	160	180	10					624
CCA	259	142	64	6					471
CCG	85	25	15	4					129
CCT	780	554	289	4					1627
CGA	24	3	2						29
CGC	109	49	18	1					177
CGG	84	39	10	2					135
CGT	21	4	3						28
CTA	96	52	41	8					197
CTC	752	473	221	10					1456
CTG	477	248	151	13					889
CTT	340	197	197	9					743
GAA	304	139	170	16					629

GAC	38	24	4	3	69
GAG	1076	672	275	7	2030
GAT	185	94	118	16	413
GCA	396	173	113	10	692
GCC	99	42	11	6	158
GCG	91	29	14	4	138
GCT	508	215	166	10	899
GGA	613	416	223	4	1256
GGC	96	33	13	5	147
GGT	181	117	67	9	374
GTA	90	57	27	11	185
GTC	28	13	3	3	47
GTG	136	70	40	11	257
GTT	167	77	56	5	305
TAA	598	472	350	8	1428
TAC	183	95	65	7	350
TAG	137	67	42	7	253
TAT	625	397	345	5	1372
TCA	233	127	128	16	504
TCC	678	443	222	10	1353
TCG	23	6	1	1	31
TCT	399	226	153	11	789
TGA	212	120	149	15	496
TGC	362	168	91	3	624
TGG	160	87	33	12	292
TGT	291	132	117	7	547
TTA	730	458	377	12	1577
TTC	356	174	142	7	679
TTG	232	92	53	14	391
AAAC	49	19			68
AAAG	86	10			96
AAAT	183	6			189
AACA	26	5			31
AACC	8	2			10
AACG	1	2			3
AACT	7	9			16
AAGA	51	15			66
AAGC	3				3
AAGG	18	7			25
AAGT	4				4
AATA	133	7			140
AATC	41	10			51
AATG	44	15			59
AATT	29	5			34
ACAA	27	12			39
ACAG	90	10			100

ACAT	31	6	37
ACCA	9	2	11
ACCC	1		1
ACCG	3	2	5
ACCT	3		3
ACGC	6	10	16
ACGG		1	1
ACGT	1		1
ACTA	7	6	13
ACTC	3	5	8
ACTG	20	5	25
ACTT	2		2
AGAA	58	14	72
AGAC	92	10	102
AGAT	152	10	162
AGCC	6	4	10
AGCG	1		1
AGCT	7		7
AGGA	26	14	40
AGGC	5	4	9
AGGG	14	3	17
AGGT	4	1	5
AGTA	4	2	6
AGTC	10	7	17
AGTG	11	5	16
AGTT	17	6	23
ATAA	104	10	114
ATAC	35	8	43
ATAG	230	9	239
ATCA	39	16	55
ATCC	69	13	82
ATCG	1		1
ATCT	225	9	234
ATGA	49	13	62
ATGC	3	1	4
ATGG	58	12	70
ATGT	26	4	30
ATTA	34	8	42
ATTC	29	9	38
ATTG	39	7	46
ATTT	111	9	120
CAAA	17	10	27
CAAC	8	2	10
CAAG	1		1
CAAT	19	11	30
CACG	50	4	54

CACT	20	5	25
CAGA	103	10	113
CAGC	9	5	14
CAGG	10	6	16
CAGT	35	4	39
CATA	13	3	16
CATC	90	15	105
CATG	2	1	3
CATT	28	13	41
CCAA	7	6	13
CCAG	7	4	11
CCAT	59	13	72
CCCA	1		1
CCCT	6		6
CCGA	2	1	3
CCGT	3	2	5
CCTA	2	4	6
CCTC	18	1	19
CCTG	8	8	16
CCTT	15	3	18
CGAT	3		3
CGCA	9	7	16
CGGA	3	2	5
CGGT	1	2	3
CGTC	1		1
CGTG	6	9	15
CGTT	2		2
CTAA	12	1	13
CTAC	7	1	8
CTAT	124	12	136
CTCA	16	7	23
CTCC	16	1	17
CTGA	19	10	29
CTGC	12	2	14
CTGG	5	6	11
CTGT	122	9	131
CTTA	2	3	5
CTTC	25	8	33
CTTG	1	1	2
CTTT	42	13	55
GAAA	35	16	51
GAAG	26	5	31
GAAT	23	9	32
GACA	83	13	96
GACC	1	1	2
GACG	4	2	6

GACT	19	6	25
GAGC	2		2
GAGG	25	3	28
GAGT	8	6	14
GATA	130	11	141
GATC	1		1
GATG	72	13	85
GATT	13	11	24
GCAC	24	10	34
GCAG	12	7	19
GCAT	2		2
GCCA	6	1	7
GCCG	3		3
GCCT	9	4	13
GCGA	1		1
GCGG	1		1
GCGT	7	1	8
GCTA	6	2	8
GCTC	2		2
GCTG	8	5	13
GGAA	16	9	25
GGAC	8	1	9
GGAG	42	4	46
GGAT	65	11	76
GGCA	6	3	9
GGCT	9	5	14
GGGA	17	5	22
GGGT	1		1
GGTA	3	1	4
GGTC	1	1	2
GGTG	1	1	2
GGTT	4	1	5
GTAA	6	1	7
GTAG	8	2	10
GTAT	16	2	18
GTCA	16	4	20
GTCC	4	1	5
GTCG	4	1	5
GTCT	60	8	68
GTGA	14	4	18
GTGC	31	8	39
GTGG	1		1
GTTA	8	4	12
GTTC	1		1
GTTG	7		7
GTTT	21	7	28

TAAA	102	16	118
TAAC	5	4	9
TAAG	5	1	6
TAAT	12	4	16
TACA	9	6	15
TACC	4	1	5
TACT	4		4
TAGA	202	3	205
TAGC	11		11
TAGG	8	3	11
TAGT	12	7	19
TATC	177	12	189
TATG	27	8	35
TATT	125	8	133
TCAA	23	10	33
TCAC	11	9	20
TCAG	16	2	18
TCAT	33	10	43
TCCA	87	18	105
TCCC	11		11
TCCG	5	2	7
TCCT	36	7	43
TCGC	1		1
TCGG	2		2
TCTA	161	11	172
TCTG	121	5	126
TCTT	65	6	71
TGAA	47	14	61
TGAC	25	4	29
TGAG	14	4	18
TGAT	36	8	44
TGCA	3		3
TGCC	1		1
TGCG	8	8	16
TGCT	1		1
TGGA	61	13	74
TGGC	2	1	3
TGGG	3		3
TGGT	7	2	9
TGTA	30	9	39
TGTC	62	13	75
TGTT	52	11	63
TTAA	24	7	31
TTAC	8	4	12
TTAG	10	6	16
TTAT	100	11	111

TTCA	44	10	54
TTCC	20	11	31
TTCT	39	7	46
TTGA	20	8	28
TTGC	1		1
TTGG	3	1	4
TTGT	13	5	18
TTTA	141	12	153
TTTC	62	12	74
TTTG	31	3	34
